# Additive and Interactive Associations of Environmental and Sociodemographic Factors with the Genotypes of Three Glutathione S-Transferase Genes in Relation to the Blood Arsenic Concentrations of Children in Jamaica

**DOI:** 10.3390/ijerph19010466

**Published:** 2022-01-01

**Authors:** Mohammad H. Rahbar, Maureen Samms-Vaughan, Yuansong Zhao, Sepideh Saroukhani, Sheikh F. Zaman, Jan Bressler, Manouchehr Hessabi, Megan L. Grove, Sydonnie Shakspeare-Pellington, Katherine A. Loveland

**Affiliations:** 1 Department of Epidemiology, Human Genetics and Environmental Sciences (EHGES), School of Public Health, The University of Texas Health Science Center at Houston, Houston, TX 77030, USA; Sepideh.Saroukhani@uth.tmc.edu (S.S.); Sheikh.F.Zaman@uth.tmc.edu (S.F.Z.); Jan.Bressler@uth.tmc.edu (J.B.); Megan.L.Grove@uth.tmc.edu (M.L.G.); 2 Biostatistics/Epidemiology/Research Design (BERD) Component, Center for Clinical and Translational Sciences (CCTS), The University of Texas Health Science Center at Houston, Houston, TX 77030, USA; Yuansong.Zhao@uth.tmc.edu (Y.Z.); Manouchehr.Hessabi@uth.tmc.edu (M.H.); 3 Division of Clinical and Translational Sciences, Department of Internal Medicine, McGovern Medical School, The University of Texas Health Science Center at Houston, Houston, TX 77030, USA; 4 Department of Child & Adolescent Health, Mona Campus, The University of the West Indies (UWI), Kingston 7, Jamaica; msammsvaughan@gmail.com (M.S.-V.); sydonniesp@gmail.com (S.S.-P.); 5 Department of Biostatistics & Data Science, School of Public Health, The University of Texas Health Science Center at Houston, Houston, TX 77030, USA; 6 Human Genetics Center, School of Public Health, The University of Texas Health Science Center at Houston, Houston, TX 77030, USA; 7 “Louis A Faillace, MD”, Department of Psychiatry and Behavioral Sciences, McGovern Medical School, The University of Texas Health Science Center at Houston, Houston, TX 77054, USA; Katherine.A.Loveland@uth.tmc.edu

**Keywords:** children, blood arsenic concentrations, glutathione S-transferase (GST) genes, food consumption, interaction, Jamaica

## Abstract

Arsenic (As) is a metalloid that has been classified as a xenobiotic with toxic effects on human beings, especially on children. Since the soil in Jamaica contains As, dietary intake is considered the main source of As exposure in Jamaicans. In addition, glutathione S-transferase (GST) genes, including *GSTT1*, *GSTP1*, and *GSTM1,* play an important role in the metabolism of xenobiotics including As in humans. Using data from 375 typically developing children (2–8 years) in Jamaica, we investigated the environmental and sociodemographic factors, as well as their possible interactions with the children’s genotype for GST genes in relation to having a detectable level of blood As concentration (i.e., >1.3 μg/L). Using multivariable logistic regression, we have identified environmental factors significantly associated with blood As concentrations that include a child’s age, parental education levels, and the consumption of saltwater fish, cabbage, broad beans, and avocado (all *p* < 0.01). Based on the multivariable analysis including gene x environment interactions, we found that among children with the Ile/Ile genotype for *GSTP1* Ile105Val, children who consumed avocado had higher odds of having a detectable blood As concentration compared to children who did not eat avocado.

## 1. Introduction

Arsenic (As) is a metalloid with both metal and nonmetal properties [1,2], which is both naturally occurring and found in environmental contaminants resulting from human activities [3]. Arsenic is a xenobiotic with toxic effects on human beings [2]. Inorganic arsenic (iAs) is rated first among a list of hazardous substances prepared by the United States (US) Environmental Protection Agency (USEPA) [4]. Although the normal range for mean blood As concentration for unexposed people living in the US is <1 μg/L, blood As concentrations in acutely toxic and fatal cases may be as high as 1000 μg/L or more. [5].

Routes of exposure to As in humans include ingestion, inhalation, and absorption through the skin [6]. Exposure to As can also occur through drinking water or eating foods that are contaminated by As-containing pesticides or soil rich in As [6]. Exposure to As through inhalation or skin absorption mainly occurs through occupational exposure to As in various industries or agricultural farming [7]. Irrespective of the route of exposure or whether it is high- or low-level exposure, As is carcinogenic in humans [7,8]. In fact, there is evidence suggesting that As exposure can adversely impact the physiology of the respiratory, immune, gastrointestinal, genitourinary, reproductive, and nervous systems in humans [9,10,11].

In 2008, the World Health Organization (WHO) reported that more than 200 million individuals living in 70 different countries were drinking water contaminated with iAs >10 μg/L [12]. High concentrations of As are naturally present in the groundwater of many countries, including Argentina, Chile, Mexico, China, Taiwan, India, Bangladesh, and some parts of the US [2,9]. According to McClintock et al., about 4.5 million people in Latin America drink As-contaminated water [12]. Similarly, in island nations such as Jamaica, the source of human As exposure is mainly from agricultural soil and water [13]. Another crucial source of As in Jamaica is the consumption of fish, which is a traditional and available food for people in Caribbean countries including Jamaica [14]. The average per capita fish consumption in Jamaica was estimated to be about 27.1 kg/year in 2016 [15], which was more than the world per capita food fish supply of 19.7 kg/year in 2016 [16]. Despite the numerous health advantages, fish consumption is also associated with the risk of exposure to As and mercury [14]. For example, Ricketts et al. reported that As concentrations in fish from Jamaica were from 0.11 to 7.80 μg/g [14]. Geochemical investigations also reported that Jamaican soil is rich in heavy metals, especially As, compared to the world average [17,18]. Aside from natural events, As is included in Jamaica’s environment due to some industrial activities such as the use of organometallic chemicals, insecticides, and herbicides [19].

Although drinking water is the primary source of exposure to As, the consumption of grains, vegetables, meats, fish, and foods cultivated or treated with water containing As is also a significant source of As exposure in humans [7]. For example, according to a report from the Total Dietary Study conducted by the USFDA, food contributes to 93% of total As intake [20]. Another study showed that rice and marine foods are the primary sources for As exposure in a study of children living in Spain [21]. There is also evidence that fish, beans, grains, and vegetables from Latin American countries contain high As levels [12]. In a study from Jamaica, Antoine et al. found mean As concentrations in food ranged from 0.001 mg/kg fresh weight in cabbages to 0.104 mg/kg fresh weight in bananas [18]. A study conducted in Bangladesh found uncooked rice contained higher As levels than uncooked vegetables [22].

Children may be exposed to As during the prenatal [23], perinatal [8], and postnatal periods [24]. As a neurotoxic metalloid, As is able to cross the human placenta and gather in the fetal tissues and may affect neurodevelopment [23,24,25]. It has been reported that As exposure during pregnancy is associated with decreased fetal head circumference [26]. Recent studies also showed associations between exposure to iAs and impaired brain development and cognitive dysfunction in children [4,10,25]. Some recent studies have also suggested a potential link between exposure to iAs and the diagnosis of autism spectrum disorder (ASD) [4]. Other epidemiologic studies reported that As exposure during the early years of life or in intrauterine life is associated with a long latency period for lung carcinoma and other chronic diseases [27].

Similar to other countries, drinking water, and agricultural soil that affect the quantity of As in food such as fruits, root vegetables, and grains, as well as fish and seafood are the most important sources of As exposure among Jamaican children [13]. According to the FDA in the US and the European Food and Safety Authority (EFSA), rice, rice syrups, fruits, fruit drinks, and other cereals have considerable quantities of As. Children less than three years old who ingest high amounts of rice products are considered at risk for the toxic effects of As exposure because of their developing physiological systems including neurodevelopment [7]. Similarly, another study from Bangladesh reported that the total daily intake of As through food was higher among children (4.75 × 10^−3^ mg per kg of the average body weight per day) in comparison with adults (2.59 × 10^−3^ mg per kg of the average body weight per day) [28]. Bangladesh and Jamaica are both low to middle-income countries with similar socioeconomic conditions except for food including the consumption of fish and seafood. Therefore, considering that fish is the main part of the Jamaican diet, high exposure to As through dietary intake is highly possible in Jamaican children. There is also evidence that Jamaican children have exposure to As through food, water, and environmental contaminants. For example, findings from our previous studies showed that blood As concentrations in Jamaican children are about four times higher than in children in the US and Canada [13]. We have also previously reported an association of glutathione-S-transferase (GST) metabolic genes and blood As concentrations in Jamaican children [29]. Hence, since genetic factors are non-modifiable, As exposure reduction should focus on dietary and environmental factors.

Human GST genes, including glutathione-S-transferase pi 1 (*GSTP1*), glutathione-S-transferase mu 1 (*GSTM1*), and glutathione-S-transferase theta 1 (*GSTT1*), play a crucial role in As metabolism [30]. These GST genes encode a family of phase II enzymes called glutathione transferases that facilitate the excretion of xenobiotics (e.g., As) by catalyzing their conjugation with the reduced form of glutathione (GSH) [31,32]. These genes have an important role in detoxification through participation in two pathways (oxidative methylation and reductive methylation) for AS metabolites (As^III^, arsenate (As^V^), monomethylarsonic acid (MMA^V^), and dimethylarsinic acid (DMA^V^)) and *GSTP1* is involved in the reduction pathway [33,34]. Marcos et al. reported the level of % DMA^V^ was higher in individuals with the *GSTP1* Ile105Val Val/Val genotype, but the difference was not significant statistically [35]. GST genes are highly polymorphic [36]; for example, null alleles of GST genes such as *GSTT1* and *GSTM1* can lead to a lack of functional enzymes and result in a decrease in detoxification capacity and cell protection from oxidative stress [37,38]. The null genotypes for *GSTT1* and *GSTM1* have been suggested as genetic factors responsible for inter-individual differences in As metabolism [30]. For example, previous studies have shown that the *GSTM1* null genotype impedes As methylation, which results in increased As retention [12].

Since 2009, our research team at the University of Texas Health Science Center at Houston (UTHealth) has been investigating the additive and interactive associations of six heavy metals, including As and GST genes (*GSTP1*, *GSTT1*, and *GSTM1*) among Jamaican children in collaboration with faculty at the University of the West Indies (UWI), Mona campus, in Jamaica. Using data from 100 (age 2–8 years) 1:1 age and sex-matched ASD cases and typically developed (TD) controls, we previously investigated the association between blood As concentrations and GST genotypes among Jamaican children with or without ASD. Our findings indicated a significant interaction between *GSTP1* and ASD case status in relation to blood As concentration in Jamaican children after controlling for several confounding variables (*p* = 0.04). Specifically, based on recessive genetic models, TD children with the Ile/Ile or Ile/Val genotype for *GSTP1* had significantly higher geometric mean blood As concentrations than those with the Val/Val genotype (3.67 μg/L vs. 2.69 μg/L, *p* < 0.01). Although, among the ASD cases, this difference was not statistically significant (3.71 μg/L vs. 3.29 μg/L, *p* = 0.29), the direction of the observed difference was consistent with that of the TD control children. These findings suggested a possible role of *GSTP1* in the detoxification of As that may influence the risk of As-related disease and disorders in Jamaican TD children [29]. We also found that Jamaican children living in Kingston parish had higher total blood As concentrations compared to those in other parishes. In addition, we found that eating avocado, callaloo, broccoli, pak choi, and not using piped water for drinking purposes was significantly associated with higher blood As concentrations in children regardless of their ASD status [13]. In this present research, we assessed the relationship of environmental and sociodemographic factors, as well as the possible interactions of these factors with genotype for GST genes, with having detectable blood As concentrations in Jamaican TD children.

## 2. Materials and Methods

### 2.1. General Description

We used data from TD Jamaican children aged 2–8 years old who were enrolled as the controls in the Epidemiological Research on Autism in Jamaica (ERAJ) and ERAJ-Phase2 (ERAJ-2) age-and sex-matched studies of ASD. The main goal of the ERAJ studies was to investigate if environmental exposures and three GST genes (*GSTP1*, *GSTT1*, and *GSTM1*) have potential additive or interactive associations in relation to ASD status in Jamaican children; detailed information regarding the recruitment and assessment of ASD cases and TD controls has been reported previously [39,40,41]. Relevant to the research objectives here, TD controls were within six months of the age-matched ASD case and were identified from schools and well-child clinics. To rule out developmental disorders in the TD children, the Social Communication Questionnaire (SCQ) [42] was administered to the parents/guardians of potential TD controls. Only children with SCQ scores ≤6 were considered as TD controls. We also administered a socioeconomic status (SES) questionnaire to assess demographic characteristics, parents’ educational levels and the family’s SES that was measured by the ownership of a car by the family. In addition, a food frequency questionnaire was used to collect information representative of dietary sources consumed by the children on a weekly basis. This included the types of seafood, organ/meat, grain/starches, beans, vegetables, and fruits classified based on their characteristics and species. For example, types of seafood were classified into the following categories: saltwater fish, freshwater fish (pond fish, tilapia), sardine or mackerel (canned fish), canned tuna, salted fish (pickled mackerel), shellfish (lobsters, crabs), and shrimp. Information was also collected regarding the sources of drinking and cooking water. For analysis, the frequency of food consumption was categorized into binary variables (consumed vs. never consumed). Details regarding the categories of foods have been reported earlier [43]. At the end of the interview and all other assessments, 4–5 mL of whole blood was collected from each child to assess exposure to the heavy metals including As and to determine GST gene genotypes. This study was approved by the Institutional Review Boards of the Michigan Department of Health and Human Services (MDHHS), UTHealth, and the UWI, Mona campus, in Kingston, Jamaica. In this study, we used data from 375 TD control children who were enrolled in the ERAJ studies between December 2009 and September 2017.

### 2.2. Assessment of As Exposures

Total blood As concentrations were assessed at the Trace Metals Lab, a Centers for Disease Control and Prevention (CDC)-certified lab at the MDHHS in Lansing, Michigan, USA. All samples were diluted and analyzed on a PerkinElmer Elan DRC II inductively coupled plasma mass spectrometer (PerkinElmer, Waltham, MA, USA). Specifically, MDHHS used method number ITB001A (Environmental Health Method: Blood Lead and Cadmium ICPDRCMS) that is based on the CDC guidelines. (https://www.cdc.gov/nchs/data/nhanes/nhanes_05_06/pbcd_d_met_lead_cadmium.pdf, accessed on 20 August 2021) Furthermore, MDHHS is a College of American Pathologists (CAP) and Clinical Laboratory Improvement Amendments 1988 (CLIA’88) accredited laboratory, and follows the quality control (QC) plan established in these guidelines. For QCs, MDHHS purchased Seronorm^TM^ Trace Elements Whole Blood which was characterized following accreditation guidelines. Once characterized, ±3SD ranges were established for each analyte at each QC level, and the QCs must meet these established criteria in order to accept the run [44].

Since the technology to detect metal concentrations in blood samples has changed over the last 11 years, different limits of detection (LoD) for As were used by MDHHS in phases 1 and 2 of the ERAJ study (LoD for As was 1 μg/L in phase 1 and 1.3 μg/L in phase 2). In this study that includes data from both phases 1 and 2, MDHHS reported 41% of blood As concentrations as undetectable; that is, they were below the LoD.

### 2.3. Genetic Analysis

Whole blood was processed and stored by the CARIGEN lab at UWI and later shipped to the UTHealth School of Public Health (UTSPH) Human Genetics Center (HGC) Laboratory in Houston, Texas. All procedures used for genetic analysis were conducted as described previously [39,40]. In brief, regions of the *GSTM1* and *GSTT1* genes were amplified in two independent TaqMan Copy Number Assay reactions: *GSTM1* Assay ID: Hs02575461_cn and *GSTT1* Assay ID: Hs00010004_cn (www.thermofisher.com, accessed on 20 August 2021). *GSTM1* and *GSTT1* homozygous deletions were coded as DD and the presence of an insertion was coded as I* to detect insertion/deletion polymorphisms. The *GSTP1* Ile105Val polymorphism (rs1695) was genotyped using the TaqMan Drug Metabolism SNP Genotyping Assay C_3217198_20.

### 2.4. Statistical Analysis

Descriptive analyses were conducted to assess the distributions of demographic and socioeconomic status (SES) characteristics of TD children. Since a sizeable portion of concentrations were below the LoD for As, we converted blood As concentrations to a binary variable using 1.3 μg/L as the cutoff point. The choice of cutoff point reflects the LoD in Phase 2 of the ERAJ study.

For *GSTT1* and *GSTM1*, since the genotyping assay does not distinguish between a normal homozygote (I/I) and a heterozygote (I/D), the recessive genetic model is the only model we considered using a binary variable: I/* (I/I or I/D) and DD (null). For the *GSTP1* Ile105Val polymorphism, there are three common genotypes (Ile/Val, Ile/Ile, Val/Val) and all genotypes were available for analysis. Hence, we analyzed the *GSTP1* genotypes using different genetic models, including dominant (Ile/Ile vs. Ile/Val or Val/Val), co-dominant (Ile/Ile, Ile/Val and Val/Val) and recessive models (Ile/Ile or Ile/Val vs. Val/Val). Moreover, we tested whether the *GSTP1* polymorphism met the Hardy–Weinberg equilibrium expectations using the chi-square test based on information for the TD children.

We used univariable logistic regression models to investigate the possible additive association of various exposures including the three GST genes, socio-demographic characteristics, and the consumption of various types of food in relation to binary blood As concentrations. We used multivariable logistic regression models to evaluate the potential gene–environment interactions between each of the three GST genes and various environmental factors in relation to binary blood As concentrations (<LoD vs. ≥LoD). Subsequently, additive and interactive models were fit to evaluate the relationship between environmental factors and having a detectable level of As concentration in blood. In order to minimize potential effects of multicollinearity, we initially checked pairwise correlations between all pairs of individual environmental factors that were statistically significant in the additive models and only kept one of the correlated variables when the model became unstable by adding both correlated variables. When we found significant gene–environmental interactions, odds ratios and 95% confidence intervals for the association between children’s exposure to environmental factors and blood As concentrations by children’s genotypes for GST genes were calculated using the CONTRAST statement in SAS [45]. We also calculated odds ratios and 95% confidence intervals for the associations between children’s genotypes for GST genes and blood As concentrations by exposure to environmental factors. All statistical tests were performed at the 0.05 significance level without making any adjustments for multiple comparisons. SAS 9.4 was used for all statistical analysis [46].

## 3. Results

A total of 375 TD children in Jamaica participated in this study. Table 1 presents the demographic information and other characteristics of children and their parents. About 25% of the TD children were 72 months or older and 81.9% were male. Nearly all of the children (97.3%) were Afro-Caribbean and 61.9% were born in Kingston parish. At the time of the child’s birth, 11.6% of mothers were age ≥35 years old and 45.5% of the children had at least one parent with an education level beyond high school. Regarding the SES, 40.3% of the families had a high SES, which is measured by the car ownership of the parents or family. For TD children, the frequency of the *GSTT1* and *GSTM1* null genotypes were 25.8% and 24.9%, respectively. Moreover, the frequencies of the *GSTP1* genotypes in the TD children were in agreement with the Hardy–Weinberg equilibrium’s expectations (*p* = 0.77).

In the univariable logistic regression models, the odds of having a detectable blood As concentration (i.e., above LoD) in children who were 72 months old or greater was 2.51 times higher than that of children younger than 72 months (OR (95% CI): 2.51 (1.49, 4.22), *p* < 0.01). Children who had at least one parent with an education level beyond high school had lower odds of having a detectable blood As concentration compared to those whose parents had not attained this level (OR (95% CI) = 0.43 (0.28, 0.66), *p* < 0.01). Similarly, children who had families with a high SES had lower odds of having a detectable blood As concentration compared to those whose families had lower SES (OR (95% CI) = 0.63 (0.42, 0.96), *p* = 0.03). In addition, children who exhibited pica (i.e., placing mud in their mouth) had higher odds of having a detectable blood As concentration compared to children who did not have such habits (OR (95% CI) = 3.56 (1.19, 10.69), *p* = 0.02). When we assessed associations between dietary consumption and blood As concentrations, we observed that children who reported eating pasta, macaroni, or noodles had lower odds of having a detectable blood As concentration compared to children who never ate such foods (OR (95% CI) = 0.46 (0.24, 0.90), *p* = 0.02). Similar results were found in relation to the consumption of cabbage and having a detectable blood As concentration (OR (95% CI) = 0.56 (0.36, 0.86), *p* = 0.01). In addition, the odds of having blood As concentrations above LoD were higher in children who consumed saltwater fish (OR (95% CI) = 2.25 (1.44, 3.52), *p* < 0.01], freshwater fish (OR (95% CI) = 1.71 (1.08, 2.69), *p* = 0.02), canned fish (sardine and mackerel) (OR (95% CI) = 1.89 (1.08, 3.30), *p* = 0.03), and organ/meats (e.g., liver) (OR (95% CI) = 2.04 (1.33, 3.12), *p* < 0.01) than in children who did not consume those foods including organ/meats. Moreover, children who consumed other beans, vegetables and fruits (peas, beans, lettuce, callaloo, string beans, tomatoes, and avocado), grain and starches (fried dumpling, and cakes/buns) had higher odds of having a detectable blood As concentration compared to those who never consumed such foods (all *P* < 0.01). There were no significant associations between blood As concentrations and *GSTM1*, *GSTP1*, and *GSTT1* genotypes (all *p* > 0.05). Findings of associations between blood As concentrations and other environmental factors are shown in Table 2.

We also assessed the relationship between environmental exposures and binary blood As concentrations by children’s genotypes for GST genes using unadjusted interactive multivariable logistic regression models that included the interaction between GST genes and the environmental exposure in relation to blood As concentrations (Table 3).

In a recessive genetic model for *GSTM1*, we have identified a significant interaction between a child’s gender and a child’s genotypes for *GSTM1* in relation to blood As concentrations (overall interaction *p* = 0.02). Specifically, among children with the DD genotype, boys were 3.75 times more likely than girls to have a detectable blood As concentration (OR (95% CI) = 3.75 (1.18, 11.94), *p* = 0.03), whereas, among children with I/I or I/D genotypes, there was no significant association between a child’s gender and blood As concentrations (*p* = 0.39). When considering *GSTP1* genotypes, we found a significant interaction between the parish of a child’s birth and the children’s genotypes for *GSTP1* in relation to a detectable level of blood As concentration using a co-dominant genetic model (overall *p* = 0.01). Specifically, although there was no significant association between the parish of a child’s birth and blood As concentrations among children with the Ile/Ile or Val/Val genotypes (*p* = 0.45 and *p* = 0.51, respectively), in children with the Ile/Val genotype, the odds of having detectable blood As concentrations were higher in children born in Kingston parish than those born in other parishes (OR (95% CI) = 2.76 (1.50, 5.80), *p* <0.01). In a dominant model for *GSTP1*, we also found significant interactions between children’s genotypes and parental education level (overall interaction *p* = 0.02), the consumption of ackee (overall *p* = 0.03), and the consumption of avocado (overall interaction *p* = 0.03) in relation to blood As concentrations. Specifically, among children with Val/Val or Ile/Val genotypes, children whose parents had not attained high school education were more likely to have a detectable blood As concentration compared to children who had at least one parent with an education beyond high school (OR (95% CI) = 3.31 (1.96, 5.57), *p* <0.01), whereas no statistically significant association between parents’ education level and blood As concentrations were found in children with the Ile/Ile genotype (*p* = 0.88). Furthermore, in a dominant model for *GSTP1*, we found that among children with the Ile/Ile genotype, children who ate avocado had 7.04 times the odds of having a detectable blood As concentration compared to children who never ate avocados (95% CI: (2.85, 17.37), *p* < 0.01), and children with Val/Val or Ile/Val genotypes who ate avocado had 2.28 times the odds of having a detectable blood As concentration compared to children with the same genotype who never ate avocado (95% CI: (1.37, 3.81), *p* < 0.01). Additional details regarding the unadjusted associations between children’s genotypes for GST genes and blood As concentrations by environmental factors are also shown as part of the Supplementary Materials (Table S1).

Adjusted associations between children’s exposure to environmental factors and blood As concentrations are described in Table 4. In the final additive multivariable model, we found that a child’s age, parental education level, and consumption of saltwater fish, cabbage, beans and avocado were significantly associated with having a detectable blood As concentration (all *p* ≤ 0.01). For example, children who eat avocado still had significantly higher odds of having a detectable blood As concentration compared to children who never ate avocado (OR (95% CI) = 2.18 (1.32, 3.60), *p* < 0.01). When we entered the interaction between the GST gene and environmental factors into the model, we found a significant interaction between *GSTP1* and the consumption of avocado using either a dominant or co-dominant genetic model (overall *p* = 0.004 and *p* = 0.01, respectively). In addition, in these interactive multivariable models, the child’s age, parental education level, and the consumption of saltwater fish, cabbage and beans were identified as other environmental factors associated with blood As concentrations in TD Jamaican children (all *p* ≤ 0.01 for both adjusted models).

Specifically, in a dominant genetic model for *GSTP1* in addition to the aforementioned environmental factors that were significantly associated with blood As concentrations, we found that among children with the Ile/Ile genotype, the odds of having detectable blood As concentrations in children who ate avocado was 7.43 times higher than those who never ate avocados (95% CI: (3.77, 20.07), *p* < 0.01); however, these associations were not significant among children with Val/Val or Ile/Val genotypes (*p* = 0.35). We observed similar findings using the co-dominant genetic model for *GSTP1*. Specifically, in addition to the aforementioned environmental factors that were significantly associated with blood As concentrations, we found that the association between the consumption of avocado and blood As concentrations was significant among children with the Ile/Ile genotype (OR (95% CI) = 7.44 (2.75, 20.10), *p* < 0.01), whereas, it was non-significant among children with Val/Val or Ile/Val genotypes (*p* = 0.27 and *p* = 0.66, respectively).

Details about adjusted associations between children’s genotype and blood As concentrations are shown in Table S2.

## 4. Discussion

In this study, we have examined the environmental and sociodemographic factors, as well as their possible interactions with children’s genotypes for GST genes in relation to having a detectable blood As concentration (above LoD) in Jamaican TD children. In an additive multivariable model, we found that although the consumption of saltwater fish, avocado, and beans was associated with about 2–3 times higher odds of having a detectable blood As concentration, the consumption of cabbage was associated with 50% lower odds of having a detectable blood As concentration in Jamaican TD children. In addition, we found that the odds of having a detectable blood As concentration in children 72 months and older and in those whose parents had education levels up to high school were 2.27 and 1.82 times higher than in younger children or children with parents who have higher education levels, respectively. In interactive multivariable models, we also found a significant interaction between children’s genotype for *GSTP1* and the consumption of avocado in relation to having a detectable blood As concentration using either dominant or co-dominant models (overall interaction *p* = 0.004 and *p* = 0.01, respectively). Specifically, after controlling for the child’s age, parental education levels, and consumption of saltwater fish, cabbage, and broad beans, we found that the odds of having a detectable blood As concentration among children with the Ile/Ile genotype who ate avocado was 7.44 and 7.43 times that of those with the same *GSTP1* genotype who never ate avocados in the co-dominant and dominant genetic models, respectively. In the following, we discuss each of these main findings separately.

### 4.1. Association of Seafood Consumption and Blood As Concentrations

Our findings that suggest 2–3 time higher odds of having detectable blood As concentrations among children who consumed fish as compared to those who did not eat fish is consistent with those of a study in the Mediterranean area that found As concentrations in cord blood were significantly associated with the frequencies of total fish consumption (r(s) = 0.350, *p* < 0.001) [47], as well as another study in Shanxi, China that reported a direct association between fish consumption and blood As concentrations in women (*p* < 0.05) [48]. Although fish is an important source of high-quality nutrients such as omega-3 fatty acids, which have been shown to prevent cardiovascular disease [49], improved brain development [50], and may even prevent certain cancers [51], it is also considered one of the main contributors to the total dietary intake for As in many populations [52,53,54,55]. For example, a study in North Italy has reported that fish and seafood were the top contributors to As exposure by evaluating trace element content in foods and their related usual dietary intake [54]. As mentioned earlier, fish consumption is an essential part of the traditional diet in Jamaica as the third-largest island country located in the Caribbean Sea [14]. Therefore, exposure data for toxic metals and metalloids including As through fish consumption should be useful for the public health authorities in order to raise awareness about these important issues and the potential concern in their country. Our results contribute to the body of knowledge on seafood consumption and blood As concentrations in Jamaican children, who are one of the populations most vulnerable to the adverse effects of exposure to As, especially on neurodevelopment.

### 4.2. Consumption of Fruits, Vegetables and Blood As Concentrations

Jamaica has high levels of As in soils [56,57,58] as well as surface water [17]. Since the ability to absorb As in soil and water differs between plants [58,59,60], the food safety of agricultural plants is of concern. In our pervious study, we reported that Jamaican children who ate avocado had a geometric mean blood As concentration of 4.78 μg/L, which is 10% higher than that among Jamaica children who did not eat such food (*p* = 0.04) [13]. In addition, Yañez et al. have reported that broad beans accumulate As effectively and highlighted that when both the soil and the irrigation water contains high levels of As, eating broad beans could pose a greater risk of having a higher level of exposure to As [60]. All these studies support our results that the consumption of broad beans and avocado is directly associated with a detectable level of blood As concentrations in Jamaican children.

Another finding in our multivariable additive analysis suggested an inverse association between the consumption of cabbage and blood As concentrations. This finding is consistent with a previous study by Antoine et al. that reported that the As content is 0.001 mg/kg in cabbage, which is the lowest in 13 Jamaican-grown food crops [18]. Another possible explanation for our finding is that eating cabbage may be correlated with having different dietary patterns and choices of food with different As content that may contribute to the blood As concentrations in Jamaican children.

### 4.3. Role of GST Genes in Blood As Concentrations of Jamaican Children with and without the Consumption of Avocado

One of the unique aspects of our study is the availability of data related to children’s genotypes for *GSTT1*, *GSTM1*, and *GSTP1* genes. In an additive model, we did not find a significant association between GST gene polymorphisms and blood As concentrations. However, investigating the possible interactive effects of *GSTP1* genotypes and the consumption of avocado in relation to blood As concentrations in Jamaican children after controlling for environmental factors, we found that *GSTP1* could be an effect modifier for the association between the consumption of avocado and blood As concentrations when using either a co-dominant or dominant genetic model. Specifically, our findings suggest that having at least one Val allele for the *GSTP1* Ile105Val polymorphism is associated with a more effective detoxification of As in Jamaican children who ate avocados. The specifics of the As metabolic pathway are complicated and not currently well understood [61]. There is evidence suggesting that the detoxification of both inorganic and organic As in humans depends on their conjugation with glutathione (GSH), a mechanism that relies on GST enzymes [61] and polymorphisms in glutathione-related genes [62,63]. According to previous studies in mice and in vitro, glutathione reductase (GR), an enzyme that maintains the supply of GSH, has been shown to be potentially inhibited by both arsenate and its methylated metabolites [64,65,66,67]. In other words, the metabolism of As would compromise the antioxidant mechanisms by consuming GSH and decreasing the GSH pool through inhibiting GR [64]. Since avocado is naturally rich in GSH [68,69] and might contain As, it is possible that there is a joint effect of the consumption of avocado and GST genes in relation to blood As concentrations. However, evidence from human studies is limited. A study from Bangladesh [70] reported that through binding and irreversible loss in bile and/or possibly in urine, concentrations of GSH and other nonprotein sulfhydryls may be influenced by As. In our previous study in Jamaica, higher blood arsenic concentrations were found in TD children with either an Ile/Ile or Ile/Val *GSTP1* genotype compared to TD children with the genotype Val/Val. Although the association was not statistically significant, we have found a similar direction of the observed difference among the ASD cases [29]. To our knowledge, we are the first to report an interactive association of *GSTP1* Ile105Val and the consumption of avocado in relation to blood As concentrations in Jamaican TD children. The confirmation of this finding in other populations is warranted.

### 4.4. Role of Parental Education in Blood As Concentrations

Our study has shown that parental education level is inversely related to children’s blood As concentrations. Since parental education, as well as income (measured by car ownership in Jamaica), are important determinants of SES [71], this finding may be attributable to the overall SES of families that can also affect dietary patterns. Several studies have shown that people with a lower SES are more vulnerable to exposure to chemical contaminants, including As [72,73,74]. For example, a study from Bangladesh reported that As-related skin lesions are more likely among people with a lower SES [74]. It is also possible that people with a higher SES have more chance and ability to take protective actions to avoid potential As exposure. For example, a study regarding As exposure in private well water showed that well owners with higher SES have more safety concerns about the quality of the untreated water, hence they are more willing to test their wells regularly to prevent the risk of possible As exposure than lower SES well owners [75]. In our study, parents with higher education levels may have had more knowledge about the possible harmful effects of As which may have influenced the food choices for their children to avoid or minimize potential exposure to As.

## 5. Limitations

The first limitation in this study is that the findings reported here may not be generalizable to all children in Jamaica because the participants were TD control children in the ERAJ study who were selected to match the ASD cases by sex and age and more likely to be from the Kingston area. Second, we acknowledge that using urine samples in which different species resulting from organic and inorganic arsenic exposure can be distinguished, particularly for assessing As metabolites, may have provided more comprehensive findings. [76]. In addition, our data cannot distinguish which *GSTP1* genotype affects detoxification by the two As pathways because we do not have detailed information about the metabolism of As, and a detailed discussion about the distinct sources of As exposure is not available due to limited resources. Furthermore, the food frequency questionnaire that we used to collect information about the consumption of food by children in the ERAJ study does not distinguish the frequency of the consumption of each type of fish separately. For instance, we have data on the number of “saltwater fish” servings that children ate per week but did not have detailed information about each type of saltwater fish consumed per week. Thus, the lack of detailed information about subgroups limited our ability to analyze the individual associations of each type of fish or seafood with blood As concentrations in children. We also found that the consumption of some food items including peas, leafy vegetables (lettuce, callaloo, broccoli, or pakchoi), string beans, and tomatoes was positively associated with blood As concentrations in the univariable models. However, these food items were removed from the multivariable analysis to avoid the potential for multicollinearity among these, possibly due to correlated independent variables in the regression model. Since fruits and vegetables are important sources of many nutrients including vitamins, fiber and phytochemicals [77], we recommend further research focused on the assessment of the risks and benefits of the consumption of each type of fruit and vegetable in Jamaica. Although several studies have shown that the source of drinking water is an important As source in human activity [2,9,78], our study did not find any significant association between drinking water source and a detectable blood As concentration in Jamaican children. Specifically, since the frequency of using ‘piped water’ as a drinking water source is at least 95% in each group, it was not possible to evaluate the relationship between drinking water source and having a detectable blood As concentration in this study. A similar issue was found in investigating the role of eating rice in relation to blood As concentrations in Jamaican children. In addition, it is possible that some participants may have consumed foods imported from other locations. However, our analysis did not assess this possibility in the food frequency questionnaire which may violate the assumption that most products were grown and caught locally. Therefore, our findings should be replicated in other populations.

## 6. Conclusions

In this study, we have identified significant environmental factors associated with blood As concentrations including a child’s age, parental education levels, and consumption of saltwater fish, cabbage, broad beans, and avocado. In addition, based on the multivariable analysis including the gene × environment (G × E) interaction, we have reported that among children with the Ile/Ile genotype for *GSTP1* Ile105Val, children who consumed avocado had higher odds of having a detectable blood As concentration compared to children who do not eat avocado. This finding suggests that having at least one Val allele for the *GSTP1* Ile105Val polymorphism is associated with a more effective detoxification of As in Jamaican children who ate avocados. We believe increasing awareness among parents regarding how these dietary and environmental factors could potentially lead to a lower level of As exposure in Jamaican children, especially among those who are more susceptible to adverse outcomes of As exposures due to their genetic variants.

## Figures and Tables

**Table 1:** Demographic and socioeconomic characteristics of children and their parents (*n* = 375).

Variables	Categories	*n* (%)
Child’s sex	Male	307 (81.9)
	Female	68 (18.1)
Child’s age (months)	Age < 72	281 (74.9)
	Age ≥ 72	94 (25.1)
Child’s race	Afro-Caribbean	365 (97.3)
Parish of child’s birth	Kingston parish	232 (61.9)
	Other parishes ^a^	143 (38.1)
Maternal age (at child’s birth) ^b^	Age < 35	326 (88.4)
	Age ≥ 35	43 (11.6)
Parental education level ^c^	Both up to high school ^d^	199 (54.5)
	At least one beyond high school ^e^	166 (45.5)
Socioeconomic status (SES)	High SES (own a car)	151 (40.3)
*GSTT1* ^f^	DD ^i^	92 (25.8)
	I/I or I/D ^j^	264 (74.2)
*GSTM1* ^g^	DD ^i^	89 (24.9)
	I/I or I/D ^j^	268 (75.1)
*GSTP1* ^h^	Ile/Ile	96 (26.7)
	Ile/Val	182 (50.7)
	Val/Val	81 (22.6)

^a^ Include Portland, Trelawny, Westmoreland, Clarendon, St. Andrew, St. Mary, St. James, St. Elizabeth, St. Catherine, St. Thomas, St. Ann, Hanover, or Manchester. ^b^ Maternal age was missing for six mothers. ^c^ Parental education level was missing for ten parents. ^d^ Up to high school education means attended primary/jr. secondary, and secondary/high/technical schools. ^e^ Beyond high school education means attended a vocational, tertiary college, or university. ^f^ *GSTT1* was missing for 19 children. ^g^ *GSTM1* was missing for 18 children. ^h^ *GSTP1* was missing for 16 children. ^i^ DD indicates the null alleles for *GSTT1* and *GSTM1*. ^j^ I/I or I/D indicate the homozygote (I/I) or a heterozygote (I/D) for *GSTT1* and *GSTM1*.

**Table 2:** Associations of environmental factors and children’s genotypes for GST genes with blood As concentrations based on univariable logistic regression models (*n* = 375).

Exposure Variables	Categories		≥LoD (*n* = 221)	<LoD (*n* = 154)	OR (95% CI)	*p* Value ^a^
Child’s gender	Male		183 (82.8)	124 (80.5)	1.17 (0.69, 1.98)	0.57
Child’s age (months)	Age ≥ 72		70 (31.7)	24 (15.6)	2.51 (1.49, 4.22)	<0.01
Child’s race	Afro-Caribbean		216 (97.7)	149 (96.8)	1.45 (0.41, 5.10)	0.56
Place of child’s birth	Kingston parish		144 (65.2)	88 (57.1)	1.40 (0.92, 2.14)	0.12
Maternal age (at child’s birth) ^b^	Age ≥ 35		25 (11.6)	18 (11.7)	0.99 (0.52, 1.89)	0.98
Parental education level ^c^	At least one beyond high school ^d^		80 (37.0)	86 (57.7)	0.43 (0.28, 0.66)	<0.01
Socioeconomic status (SES)	High SES (own a car)		79 (35.8)	72 (46.8)	0.63 (0.42, 0.96)	0.03
*GSTT1* ^e^	DD ^g^		48 (23.2)	44 (29.5)	0.72 (0.45, 1.16)	0.18
	I/I or I/D ^h^		159 (76.8)	105 (70.5)	REF	
*GSTM1* ^f^	DD ^g^		51 (24.6)	38 (25.3)	0.96 (0.59,1.56)	0.88
	I/I or I/D ^h^		156 (75.4)	112 (74.7)	REF	
*GSTP1* ^i^	Ile/Ile		54 (25.8)	42 (28.0)	REF	
	Ile/Val		106 (50.7)	76 (50.7)	1.08 (0.66, 1.79)	0.75
	Val/Val		49 (23.5)	32 (21.3)	1.19 (0.65, 2.17)	0.57
Living near a high traffic road			80 (36.2)	65 (42.2)	0.78 (0.51, 1.18)	0.24
Pica (habitually put items in mouth)	Mud ^j^		19 (8.7)	4 (2.6)	3.56 (1.19, 10.69)	0.02
Source of drinking water ^k^	Piped water		208 (94.6)	150 (97.4)	0.46 (0.15, 1.46)	0.19
Source of cooking water ^l^	Piped water		210 (95.4)	152 (98.7)	0.28 (0.06, 1.28)	0.10
Seafood consumption	Saltwater fish		139 (76.5)	91 (59.1)	2.25 (1.44, 3.52)	<0.01
	Freshwater fish (Pond fish, Tilapia)		81 (36.6)	39 (25.3)	1.71 (1.08, 2.69)	0.02
	Sardine, mackerel (Canned fish)		194 (87.8)	122 (79.2)	1.89 (1.08, 3.30)	0.03
	Tuna (Canned fish)		87 (39.4)	48 (31.2)	1.43 (0.93, 2.22)	0.10
	Salt fish (Pickled mackerel)		181 (81.9)	114 (74.0)	1.59 (0.97, 2.61)	0.07
	Shellfish (Lobster, Crab)		29 (13.1)	19 (12.3)	1.07 (0.58, 1.99)	0.82
	Shrimp		42 (19.0)	24 (15.6)	1.27 (0.73, 2.20)	0.39
Organ/meat consumption	Liver		152 (68.8)	80 (52.0)	2.04 (1.33, 3.12)	<0.01
Grain and starches consumption	White rice or rice and peas		216 (97.7)	152 (98.7)	0.57 (0.11, 2.97)	0.50
	Fried dumpling (Festival dumpling)		188 (85.1)	116 (75.3)	1.87 (1.11, 3.14)	0.02
	Boiled dumpling		194 (87.8)	143 (92.9)	0.55 (0.27, 1.15)	0.11
	White bread		139 (62.9)	109 (70.8)	0.70 (0.45, 1.09)	0.11
	Whole wheat bread		147 (66.5)	92 (59.7)	1.34 (0.87, 2.05)	0.18
	Cakes/Buns		195 (88.2)	124 (80.5)	1.82 (1.03, 3.21)	0.04
	Porridge (cornmeal, oatmeal)		201 (90.9)	142 (92.2)	0.85 (0.40, 1.79)	0.67
	Cold breakfast cereal		177 (80.1)	124 (80.5)	0.46 (0.58, 1.63)	0.92
	Pasta, macaroni, noodles		184 (83.3)	141 (91.6)	0.46 (0.24, 0.90)	0.02
Beans	Peas, beans, nuts	Red peas, gungo peas	191 (86.4)	108 (70.1)	2.71 (1.62, 4.55)	<0.01
		Broad beans	158 (71.5)	66 (42.9)	3.34 (2.17, 5.15)	<0.01
		Peanuts, cashews	179 (81.0)	115 (74.7)	1.45 (0.88, 2.37)	0.14
Fruits and vegetables consumption	Root vegetables	Yam, sweet potato, dasheen, coco	146 (66.1)	113 (73.4)	0.71 (0.45, 1.11)	0.13
		Carrot, pumpkin	195 (88.2)	130 (84.4)	1.39 (0.76, 2.52)	0.29
	Leafy vegetables	Lettuce	153 (69.2)	81 (52.6)	2.03 (1.32, 3.11)	<0.01
		Callaloo, broccoli, or pakchoi	195 (88.2)	112 (72.7)	2.81 (1.64, 4.83)	<0.01
		Cabbage	125 (56.6)	108 (70.1)	0.56 (0.36, 0.86)	<0.01
	Legumes	String beans	120 (54.3)	42 (27.3)	3.17 (2.04, 4.93)	<0.01
	Fruit	Tomatoes	180 (81.4)	100 (64.9)	2.37 (1.48, 3.81)	<0.01
		Ackee	149 (67.4)	110 (71.4)	0.83 (0.53, 1.30)	0.41
		Avocado	158 (71.5)	71 (46.1)	2.93 (1.91, 4.51)	<0.01
		Green banana	148 (67.0)	117 (76.0)	0.64 (0.40, 1.02)	0.06
		Fried plantain	190 (86.0)	128 (83.1)	1.25 (0.71, 2.20)	0.45

OR: Odds ratio. ^a^ *p*-values are based on the Wald’s test in the univariable logistic regression models. ^b^ Maternal age was missing for six mothers. ^c^ Parental education level was missing for ten parents. ^d^ Beyond high school education means attended a vocational, tertiary college, or university. ^e^ *GSTT1* was missing for 14 children with blood As concentrations above LoD and 5 children with blood As concentrations below LoD. ^f^ *GSTM1* was missing for 14 children with blood As concentrations above LoD and 4 children with blood As concentrations below LoD. ^g^ DD indicates the null alleles for *GSTT1* and *GSTM1.* ^h^ I/I or I/D indicate the homozygote (I/I) or a heterozygote (I/D) for *GSTT1* and *GSTM1*. ^i^ *GSTP1* was missing for 12 children with blood As concentrations above LoD and 4 children with blood As concentrations below LoD. ^j^ Pica-mud was missing for two children with blood As concentrations above LoD. ^k^ Source of drinking water was missing for one child with blood As concentrations above LoD. ^l^ Source of cooking water was missing for one child with blood As concentrations above LoD.

**Table 3:** Associations between children’s exposure to environmental factors and a binary detectable level of blood As concentrations by children’s genotypes for GST genes based on the multivariable logistic regression models that include the interaction between GST genes and the main environmental exposure (*n* = 375).

Environmental Factor	Category Compared	Referent Category	Gene	Models	Genotypes	OR (95%CI)	*p* Value ^a^	Overall Interaction *p* Value ^b^
Child’s gender	Male	Female	*GSTM1* ^c^	Recessive	DD ^e^	3.75 (1.18, 11.94)	0.03	0.02
					I/I or I/D ^f^	0.75 (0.39, 1.44)	0.39	
			*GSTP1* ^d^	Co-dominant	Ile/Ile	0.46 (0.13, 1.60)	0.22	0.07
					Ile/Val	1.18 (0.58, 2.40)	0.64	
					Val/Val	4.29 (1.02, 18.07)	0.047	
				Dominant	Ile/Ile	0.46 (0.13, 1.60)	0.22	0.09
					Val/Val or Ile/Val	1.56 (0.84, 2.91)	0.16	
				Recessive	Val/Val	4.29 (1.02, 18.07)	0.047	0.052
					Ile/Ile or Ile/Val	0.92 (0.50, 1.68)	0.78	
Parish of child’s birth	Kingston	Other ^g^	*GSTT1* ^b^	Recessive	DD ^e^	0.74 (0.32, 1.71)	0.49	0.06
					I/I or I/D ^f^	1.91 (1.15, 3.17)	0.01	
			*GSTP1* ^h^	Co-dominant	Ile/Ile	0.71 (0.29, 1.73)	0.45	0.01
					Ile/Val	2.76 (1.50, 5.08)	<0.01	
					Val/Val	0.74 (1.83, 0.43)	0.51	
				Dominant	Ile/Ile	0.71 (0.29, 1.73)	0.45	0.07
					Val/Val or Ile/Val	1.83 (1.11, 3.02)	0.02	
				Recessive	Val/Val	0.74 (0.30, 1.83)	0.51	0.1
					Ile/Ile or Ile/Val	1.75 (1.07, 2.86)	0.03	
Parental education level ^i^	Group 1 ^j^	Group 2 ^k^	*GSTP1* ^h^	Co-dominant	Ile/Ile	1.07 (0.47, 2.41)	0.88	0.057
					Ile/Val	3.04 (1.64, 5.65)	<0.01	
					Val/Val	4.38 (1.61, 11.92)	<0.01	
				Dominant	Ile/Ile	1.07 (0.47, 2.41)	0.88	0.02
					Val/Val or Ile/Val	3.31 (1.96, 5.57)	<0.01	
				Recessive	Val/Val	4.38 (1.61, 11.92)	<0.01	0.19
					Ile/Ile or Ile/Val	2.09 (1.28, 3.42)	<0.01	
Consumption of ackee	Yes	No	*GSTP1* ^h^	Co-dominant	Ile/Ile	1.78 (0.76, 4.15)	0.18	0.09
					Ile/Val	0.66 (0.34, 1.28)	0.22	
					Val/Val	0.48 (0.17, 1.34)	0.16	
				Dominant	Ile/Ile	1.78 (0.76, 4.15)	0.18	0.03
					Val/Val or Ile/Val	0.6 (0.34, 1.04)	0.07	
				Recessive	Val/Val	0.48 (0.17, 1.34)	0.16	0.23
					Ile/Ile or Ile/Val	0.96 (0.58, 1.62)	0.89	
Consumption of avocado	Yes	No	*GSTP1* ^h^	Co-dominant	Ile/Ile	7.04 (2.85, 17.37)	<0.01	0.09
					Ile/Val	2.12 (1.15, 3.88)	0.01	
					Val/Val	2.72 (1.05, 7.05)	0.04	
				Dominant	Ile/Ile	7.04 (2.85, 17.37)	<0.01	0.03
					Val/Val or Ile/Val	2.28 (1.37, 3.81)	<0.01	
				Recessive	Val/Val	2.72 (1.05, 7.05)	0.04	0.79
					Ile/Ile or Ile/Val	3.16 (1.92, 5.20)	<0.01	

^a^ *p*-values are based on the Wald’s test in multivariable logistic regression models. ^b^ overall interaction *p*-values based on the type 3 effect test in multivariable logistic regression models. ^c^ *GSTM1* was missing for 14 children with blood As concentrations above LoD and 4 children with blood As concentrations below LoD. ^d^ *GSTT1* was missing for 14 children with blood As concentrations above LoD and 5 children with blood As concentrations below LoD. ^e^ DD indicates the null alleles for *GSTT1* and *GSTM1.* ^f^ I/I or I/D indicate the homozygote (I/I) or a heterozygote (I/D) for *GSTT1* and *GSTM1.* ^g^ Include Portland, Trelawny, Westmoreland, Clarendon, St. Andrew, St. Mary, St. James, St. Elizabeth, St. Catherine, St. Thomas, St. Ann, Hanover, or Manchester. ^h^ *GSTP1* was missing for 12 children with blood As concentrations above LoD and 4 children with blood As concentrations below LoD. ^i^ Parental education level: five missing for children with blood As concentrations above LoD, and five missing for children with blood As concentrations below LoD. ^j^ Up to high school education means attended primary/jr. secondary, and secondary/high/technical schools. ^k^ Beyond high school education means attended a vocational, tertiary college, or university.

**Table 4:** Adjusted associations between children’s exposure to environmental factors and a binary detectable level of blood As concentrations by children’s genotypes for GST genes based on multivariable logistic regression models that include the interaction between GST genes and the main environmental exposure (*n* = 375).

Models	Environmental Factor		Categories Compared	Gene	Genotypes	OR (95%CI)	*p* Value ^a^
Additive multivariable model	Child’s age (months)		Age ≥ 72 vs. Age < 72			2.27 (1.27, 4.07)	<0.01
	Parental education level ^b^		Group 1 ^c^ vs. Group 2 ^d^			1.82 (1.12, 2.97)	0.01
	Consumption of saltwater fish		Yes vs. no			1.99 (1.18, 3.34)	<0.01
	Consumption of cabbage		Yes vs. no			0.47 (0.28, 0.81)	<0.01
	Consumption of beans		Yes vs. no			2.72 (1.65, 4.48)	<0.01
	Consumption of avocado		Yes vs. no			2.18 (1.32, 3.60)	<0.01
Interactive multivariable model	Co-dominant	Child’s age (months)	Age ≥ 72 vs. Age < 72			2.41 (1.33, 4.38)	<0.01
		Parental education level ^b^	Group 1 ^c^ vs. Group 2 ^d^			2.01 (1.21, 3.32)	<0.01
		Consumption of saltwater fish	Yes vs. No			1.96 (1.15, 3.34)	0.01
		Consumption of cabbage	Yes vs. No			0.47 (0.27, 0.82)	<0.01
		Consumption of beans	Yes vs. No			2.89 (1.74, 4.82)	<0.01
		Consumption of avocado ^e^	Yes vs. No	*GSTP1* ^f^	Ile/Ile	7.44 (2.75, 20.10)	<0.01
					Ile/Val	1.17 (0.58, 2.34)	0.66
					Val/Val	1.87 (0.61, 5.75)	0.27
	Dominant	Child’s age (months)	Age ≥ 72 vs. Age < 72			2.37 (1.31, 4.30)	<0.01
		Parental education level ^b^	Group 1 ^c^ vs. Group 2 ^d^			2.01 (1.22, 3.32)	<0.01
		Consumption of saltwater fish	Yes vs. no			1.93 (1.14, 3.27)	0.01
		Consumption of cabbage	Yes vs. no			0.48 (0.28, 0.83)	<0.01
		Consumption of beans	Yes vs. no			2.92 (1.75, 4.86)	<0.01
		Consumption of avocado ^g^	Yes vs. no	*GSTP1* ^f^	Ile/Ile	7.43 (3.77, 20.07)	<0.01
					Val/Val or Ile/Val	1.33 (0.73, 2.42)	0.35

^a^ *p*-values are based on the Wald’s test in the multivariable logistic regression models. ^b^ Parental education level: five missing for children with blood As concentrations above LoD, and five missing for children with blood As concentrations below LoD. ^c^ Up to high school education means attended primary/jr. secondary, and secondary/high/technical schools. ^d^ Beyond high school education means attended a vocational, tertiary college, or university. ^e^ Overall interaction *p*-values based on the type 3 effect test is 0.01. ^f^ *GSTP1* was missing for 12 children with blood As concentrations above LoD and 4 children with blood As concentrations below LoD. ^g^ Overall interaction *p*-value based on the type 3 effect test is 0.004.

## Data Availability

The data analyzed in this study are from two grants (i.e., R21 and R01). The data from R01 are or will be publicly available through the National Database for Autism Research (NDAR) via the following link: https://nda.nih.gov/edit_collection.html?id=2063 (accessed on 14 Novemebr 2021). Data from R21 will also be available upon request from the corresponding author based on the following data sharing agreement stated in the R21 grant: (1) a commitment to using the data only for research purposes and not to identify any individual participant; (2) a commitment to using best statistical and ethical practices in analyzing and reporting findings; (3) a commitment to securing the data using appropriate information technology; (4) a commitment to crediting the source and the funding agencies of the original project in all publications and presentations; and (5) a commitment to destroying or returning the data after analyses are completed.

## Supplementary Materials

The following supporting information can be downloaded at: https://www.mdpi.com/article/10.3390/ijerph19010466/s1, Table S1: Associations between children’s genotypes for GST genes and a binary detectable level of blood As concentrations by exposure to environmental factors based on multivariable logistic regression models that include the interaction between GST genes and the main environmental exposure (*n* = 375), Table S2: Adjusted associations between children’s genotypes for GST genes and a detectable level of blood As concentrations by the exposure to environmental factors based on multivariable logistic regression models that include the interaction between GST genes and the main environmental exposure (*n* = 375).
