# Supplementary material for: Additive and Interactive Associations of Environmental and Sociodemographic Factors with the Genotypes of Three Glutathione S-Transferase Genes in Relation to the Blood Arsenic Concentrations of Children in Jamaica
Source: Int J Environ Res Public Health. 2022 Jan 1;19(1):466. doi: 10.3390/ijerph19010466 (PMC8745014; doi:10.3390/ijerph19010466)
Supplement: Supplementary file 1 [file ijerph-19-00466-s001.zip › ijerph-1488259-supplementary.pdf]

International Journal of
Environmental Research and Public Health

MDPI

Supplementary Materials

Additive and Interactive Associations of environmental and sociodemographic factors with genotypes of three glutathione S transferase genes in relation to blood arsenic concentrations of children in Jamaica

Mohammad H. Rahbar [1,2,3,*], Maureen Samms-Vaughan [4], Yuansong Zhao [2,5], Sepideh Saroukhani [1,2], Sheikh F. Zaman [1,2], Jan Bressler [1,6], Manouchehr Hessabi [2], Megan L. Grove [1,6], Sydonnie Shakspeare-Pellington [4], and Katherine A. Loveland [6]

Citation: Rahbar, M.H.; Samms-Vaughan, M.; Zhao, Y.; Saroukhani, S.; Zaman, S.F.; Bressler, J.; Hessabi, M.; Grove, M.L.; Shakspeare-Pellington, S.; Loveland, K.A. Additive and Interactive Associations of Environmental and Sociodemographic Factors with the Genotypes of Three Glutathione S-Transferase Genes in Relation to the Blood Arsenic Concentrations of Children in Jamaica. *Int. J. Environ. Res. Public Health* **2022**, *19*, 466. https://doi.org/10.3390/ijerph19010466

Academic Editor: Jitse P. van Dijk

Received: 15 November 2021
Accepted: 28 December 2021
Published: 1 January 2022

Publisher's Note: MDPI stays neutral with regard to jurisdictional claims in published maps and institutional affiliations.

Copyright: © 2022 by the authors. Licensee MDPI, Basel, Switzerland. This article is an open access article distributed under the terms and conditions of the Creative Commons Attribution (CC BY) license (https://creativecommons.org/licenses/by/4.0/).

[1] Department of Epidemiology, Human Genetics, and Environmental Sciences (EHGES), School of Public Health, The University of Texas Health Science Center at Houston, Houston, TX 77030, USA; Mohammad.H.Rahbar@uth.tmc.edu (M.H.R.); Jan.Bressler@uth.tmc.edu (J.B.); Megan.L.Grove@uth.tmc.edu (M.L.G.); Sepideh.Saroukhani@uth.tmc.edu (S.S.); Sheikh.F.Zaman@uth.tmc.edu (S.F.Z.)

[2] Biostatistics/Epidemiology/Research Design (BERD) component, Center for Clinical and Translational Sciences (CCTS), The University of Texas Health Science Center at Houston, Houston, Texas 77030, USA; Manouchehr.Hessabi@uth.tmc.edu (M.H.); Yuansong.Zhao@uth.tmc.edu (Y.Z.)

[3] Division of Clinical and Translational Sciences, Department of Internal Medicine, McGovern Medical School, The University of Texas Health Science Center at Houston, Houston, TX 77030, USA;

[4] Department of Child & Adolescent Health, The University of the West Indies (UWI), Mona Campus, Kingston, Kingston 7, Jamaica.; msammsvaughan@gmail.com (M.S.-V.); sydonniesp@gmail.com (S.S.-P.)

[5] Department of Biostatistics & Data Science, School of Public Health, The University of Texas Health Science Center at Houston, Houston, TX 77030, USA;

[6] Human Genetics Center, School of Public Health, The University of Texas Health Science Center at Houston, Houston, TX 77030, USA;

[6] "Louis A Faillace, MD", Department of Psychiatry and Behavioral Sciences, McGovern Medical School, The University of Texas Health Science Center at Houston, Houston, TX 77054, USA; Katherine.A.Loveland@uth.tmc.edu (K.A.L.)

* Correspondence: E-Mail: Mohammad.H.Rahbar@uth.tmc.edu; Tel.: +1-713-500-7901; Fax: +1-713-500-0766

Table S1. Associations between children's genotypes for GST genes and a binary detectable level of blood As concentrations by exposure to environmental factors based on multivariable logistic regression models that include interaction between GST genes and the main environmental exposure (N = 375)

Gene	Models	Genotypes Compared	Referent Genotypes	Environmental factor	Category	OR (95%CI)	*P* value [a]	Overall interaction *P* value [b]
GSTM1 [c]	Recessive	DD [e]	I/I or I/D [f]	Child's gender	Male	1.28 (0.74, 2.21)	0.37	0.018
					Female	0.26 (0.08, 0.87)	0.03	
GSTT1 [d]	Recessive	DD [e]	I/I or I/D [f]	Parish of child's birth	Kingston	0.49 (0.26, 0.93)	0.03	0.06
					Other [g]	1.27 (0.60, 2.67)	0.53	
GSTP1 [h]	Co-dominant	Ile/Val	Ile/Ile	Child's gender	Male	1.25 (0.72, 2.16)	0.42	0.07
					Female	0.49 (0.13,1.82)	0.29	
		Val/Val	Ile/Ile		Male	1.59 (0.83, 3.05)	0.16	
					Female	0.17 (0.03, 1.02)	0.052	
		Ile/Val	Val/Val		Male	0.79 (0.44,1.42)	0.43	
					Female	2.85 (0.64, 12.64)	0.17	
	Dominant	Ile/Ile	Val/Val or Ile/Val	Child's gender	Male	0.74 (0.44, 1.24)	0.25	0.09
					Female	2.5 (0.69, 9.03)	0.16	
	Recessive	Ile/Ile or Ile/Val	Val/Val	Child's gender	Male	0.72 (0.42, 1.26)	0.25	0.052
					Female	3.39 (0.79, 14.57)	0.1	
	Co-dominant	Ile/Val	Ile/Ile	Parish of child's birth	Kingston	1.88 (0.99, 3.54)	0.05	0.01
					Other [g]	0.48 (0.20, 1.16)	0.1	
		Val/Val	Ile/Ile		Kingston	1.16 (0.55, 2.46)	0.69	
					Other [g]	1.12 (0.40, 3.12)	0.83	
		Ile/Val	Val/Val		Kingston	1.61 (0.79, 3.29)	0.18	
					Other [g]	0.43 (0.19, 0.99)	0.048	
	Dominant	Ile/Ile	Val/Val or Ile/Val	Parish of child's birth	Kingston	0.62 (0.35, 1.12)	0.11	0.07

				Other [g]	1.61 (0.70, 3.71)	0.27	
Recessive	Ile/Ile or Ile/Val	Val/Val	Parish of child's birth	Kingston	1.25 (0.65, 2.41)	0.5	0.1
				Other [g]	0.53 (0.24, 1.17)	0.11	
Co-dominant	Ile/Val	Ile/Ile	Parental education level [i]	Group 1 [j]	1.73 (0.85, 3.51)	0.13	0.057
				Group 2 [k]	0.6 (0.29, 1.26)	0.18	
	Val/Val	Ile/Ile		Group 1 [j]	2.81 (1.07, 7.37)	0.04	
				Group 2 [k]	0.69 (0.29, 1.62)	0.39	
	Ile/Val	Val/Val		Group 1 [j]	0.61 (0.25, 1.49)	0.28	
				Group 2 [k]	0.88 (0.41, 1.91)	0.75	
Dominant	Ile/Ile	Val/Val or Ile/Val	Parental education level [i]	Group 1 [j]	0.51 (0.26, 1.01)	0.05	0.02
				Group 2 [k]	1.59 (0.79, 3.17)	0.19	
Recessive	Ile/Ile or Ile/Val	Val/Val	Parental education level [i]	Group 1 [j]	0.51 (0.22, 1.19)	0.12	0.19
				Group 2 [k]	1.06 (0.51, 2.18)	0.88	
Co-dominant	Ile/Val	Ile/Ile	Consumption of ackee	Yes	0.78 (0.42, 1.45)	0.44	0.09
				No	2.12 (0.88, 5.14)	0.09	
	Val/Val	Ile/Ile		Yes	0.78 (0.38, 1.63)	0.51	
				No	2.89 (0.96, 8.71)	0.06	
	Ile/Val	Val/Val		Yes	1.00 (0.53, 1.88)	0.99	
				No	0.73 (0.26, 2.09)	0.56	
Dominant	Ile/Ile	Val/Val or Ile/Val	Consumption of ackee	Yes	1.28 (0.71, 2.30)	0.42	0.03
				No	0.43 (0.19, 0.98)	0.04	
Recessive	Ile/Ile or Ile/Val	Val/Val	Consumption of ackee	Yes	1.08 (0.59, 1.97)	0.8	0.23
				No	0.54 (0.20, 1.43)	0.21	
Co-dominant	Ile/Val	Ile/Ile	Consumption of avocado	Yes	0.60 (0.29, 1.26)	0.18	0.09
				No	2.00 (0.90, 4.45)	0.09	
	Val/Val	Ile/Ile		Yes	0.69 (0.30, 1.61)	0.39	

				No	1.78 (0.66, 4.86)	0.26	
	Ile/Val	Val/Val		Yes	0.87 (0.43, 1.74)	0.7	
				No	1.12 (0.46, 2.72)	0.81	
Dominant	Ile/Ile	Val/Val or Ile/Val	Consumption of avocado	Yes	1.59 (0.79, 3.21)	0.19	0.03
				No	0.52 (0.24, 1.11)	0.09	
Recessive	Ile/Ile or Ile/Val	Val/Val	Consumption of avocado	Yes	1.02 (0.52, 1.97)	0.96	0.79
				No	0.88 (0.38, 2.04)	0.76	

[a] *P*-values are based on the Wald's test in multivariable logistic regression models. [b] Overall interaction *P*-values based on the type 3 effect test in multivariable logistic regression models. [c] *GSTM1* was missing for 14 children with blood As concentrations above LoD and 4 children with blood As concentrations below LoD. [d] *GSTT1* was missing for 14 children with blood As concentrations above LoD and 5 children with blood As concentrations below LoD. [e] DD indicates the null alleles for *GSTT1*and *GSTM1.* [f] I/I or I/D indicate the homozygote (I/I) or a heterozygote (I/D) for *GSTT1* and *GSTM1.* [g] Include Portland, Trelawny, Westmoreland, Clarendon, St. Andrew, St. Mary, St. James, St. Elizabeth, St. Catherine, St. Thomas, St. Ann, Hanover, or Manchester. [h] *GSTP1* was missing for 12 children with blood As concentrations above LoD and 4 children with blood As concentrations below LoD. [i] Parental education level: 5 missing for children with blood As concentrations above LoD, and 5 missing for children with blood As concentrations below LoD. [j] Group 1: Up to high school education means attended Primary/Jr. Secondary, and Secondary/High/Technical schools. [k] Group 2: Beyond high school education means attended a Vocational, Tertiary College, or University.

Table S2. Adjusted associations between children's genotypes for GST genes and a detectable level of blood As concentrations by exposure to environmental factors based on multivariable logistic regression models that include interaction between GST genes and the main environmental exposure (N = 375).

**Models**		**Environmental Factor**	**Category**	**Gene**	**Genotypes**	**OR (95%CI)**	***P* value [a]**
Interactive multivariable model	Co-dominant	Consumption of avocado [b]	Yes	*GSTP1*[c]	Ile/Val vs. Ile/Ile	0.49 (0.22, 1.08)	0.08
					Val/Val vs. Ile/Ile	0.57 (0.22, 1.44)	0.24
					Ile/Val vs. Val/Val	0.85 (0.39, 1.85)	0.68
			No		Ile/Val vs. Ile/Ile	3.09 (1.25, 7.64)	0.01
					Val/Val vs. Ile/Ile	2.27 (0.71, 7.30)	0.17
					Ile/Val vs. Val/Val	1.36 (0.48, 3.86)	0.56
	Dominant	Consumption of avocado [d]	Yes	*GSTP1*[c]	Ile/Ile vs. Val/Val or Ile/Val	1.96 (0.91, 4.18)	0.08
			No			0.35 (0.15, 0.83)	0.02

[a] *P*-values are based on the Wald's test in multivariable logistic regression models. [b] Overall interaction *P*-values based on the type 3 effect test is 0.01. [c] GSTP1 was missing for 12 children with blood As concentrations above LoD and 4 children with blood As concentrations below LoD. [d] Overall interaction *P*-values based on the type 3 effect test is 0.004.
